# Cellular Automaton Simulation of the Growth of Anomalous Eutectic during Laser Remelting Process

**DOI:** 10.3390/ma11101844

**Published:** 2018-09-27

**Authors:** Lei Wei, Yongqing Cao, Xin Lin, Weidong Huang

**Affiliations:** 1 State Key Laboratory of Solidification Processing, School of Materials Science and Engineering, Northwestern Polytechnical University, Xi’an 710072, China; weilei@nwpu.edu.cn (L.W.); huang@nwpu.edu.cn (W.H.); 2 School of Materials Science and Engineering, Luoyang Institute of Science and Technology, Luoyang 471000, China; suneycyq@163.com

**Keywords:** anomalous eutectic, numerical simulation, cellular automaton, solidification

## Abstract

Anomalous eutectic morphologies were observed during laser remelting of a Ni-Sn powder bed, where it was sandwiched between a lamellar eutectic at the bottom of melt pool. That is, the anomalous eutectic growth mechanism can be divided into two processes: one is the lamellar to anomalous transition (LAT); the other is the anomalous to lamellar transition (ALT). The thermal distribution at the bottom of melt pool is simulated by the finite difference method. It is found that the cooling rate at the bottom of melt pool is a linear function of time. A cellular automaton (CA) model is developed to simulate the anomalous growth. Simulation results show that the mechanism of the LAT is that one phase overgrows the other followed by subsequent nucleating of the other phase. The mechanism of the ALT is the competitive growth between the anomalous and lamellar eutectic; as the cooling rate increased, the lamellar eutectic is more competitive.

## 1. Introduction

Solidification processing [1] is important for microstructure formation [2], which determines material properties. Eutectic is one of the most commonly observed solidification patterns and has been widely investigated [3]. Eutectic morphologies can be steady-state lamellar or rod [4] and beyond [5]. Various morphological instabilities of lamellar eutectic growth have been evidenced: oscillatory patterns(1*λ*O and 2*λ*O) [6,7] and zigzag instability [8,9]. With the addition of a “ternary” component, the eutectic microstructures can be eutectic cells [10], spiral two-phase dendrites [11], and three-phase eutectic patterns [12].

Anomalous eutectic is commonly observed during solidification of binary eutectic alloy [13,14,15]. Anomalous eutectic is typically much coarser and globular than regular eutectic. It is considered that anomalous eutectic is formed by the resolidification of the remelted primary solidified structure [16]. Quantitative experiments have shown that the volume fraction of anomalous eutectic far exceeds the fraction of solid that would be expected to form during the recalescence stage of solidification [17]. To date, the morphological evolution of anomalous growth is still unknown, which is due to the lack of direct observation or simulation evidence. Regular eutectic growth has been well-investigated by various numerical models: boundary integral method [6] and phase field (PF) model [9,18]. The cellular automaton (CA) model [19] has also been used to investigate lamellar eutectic growth. To our knowledge, multiphase field simulation of Al-Si anomalous eutectic growth is one of the few that related to anomalous eutectic growth [20]. However, Si is a faceted crystal and the Al-Si alloy follows an irregular eutectic growth pattern, which is outside the scope of anomalous eutectic growth (nonfaced-nonfaced).

Anomalous eutectic growth of the Ni-Sn alloy has been observed in hypoeutectic [21], eutectic [14], and hypereutectic compositions [22]. One of the anomalous eutectic characterizations is the discontinuous *α*-Ni and continuous *β*-Ni_3_Sn, which means the morphologies of discontinuous *α*-Ni plays an important role in the formation of the anomalous eutectic. In order to avoid the influence of *β*-Ni_3_Sn nucleation, the anomalous eutectic growth of Ni-30wt.%Sn alloy was investigated. This work numerically simulated anomalous eutectic growth during laser remelting of a Ni-30wt.%Sn powder bed [21]. Simulation of anomalous eutectic growth is difficult, requiring many trial and error simulations. The CA model, which has high computational efficiency and relatively simple physical principles, has large potential for scientific and engineering simulations. Compared to the PF model, the computational efficiency in the CA model is the key aspect for us to use the CA model to simulate anomalous eutectic growth. Due to the fact that the remelting-based anomalous growth models are infeasible for anomalous eutectic growth [17], the growth mechanism of the anomalous eutectic is still unknown, and very few simulations of the growth of anomalous eutectic were published in the literatures. The temperature filed during solidification from undercooled melts is extremely complicated, that is why very few simulations are shown for this experimental condition. In the present article, the temperature field at the bottom of melt pool is derived from the thermal simulations, providing better conditions for numerical simulation, which can be simplified into directional solidification. It is shown that the cooling rate has a significant effect on the growth of the anomalous eutectic.

## 2. Materials and Methods

A eutectic CA model was developed for anomalous eutectic growth, based on the previous dendritic CA model [23].

The solute diffusion is governed by:

(1) $$\frac{\partial C_{L}}{\partial t}=D_{l}\cdot \nabla^{2}C_{l}+C_{l}(1-k_{\alpha })\frac{\partial f_{s,\alpha }}{\partial t}+C_{l}(1-k_{\beta })\frac{\partial f_{s,\beta }}{\partial t}$$

where *C_l_* is the solute concentration in liquid, *t* is time, *D_l_* is the solute diffusion coefficient in liquid, *k_α_* and *k_β_* are the partition coefficients of *α* and *β* phases, respectively, and *f_s_*_,*α*_ and *f_s_*_,*β*_ are the solid fractions. The solute diffusion in solid phase is neglected in present CA model. The governing equation is solved by an explicit finite difference method.

In the present CA model, the solid/liquid (SL) interfaces kinetics of eutectic growth is related to the local equilibrium condition:

(2) $$T_{i}^{*}=T_{E}+m_{i}(C_{l,i}^{*}-C_{E})-\Gamma_{i}\kappa_{i}f(\varphi_{i},\theta_{0,i})$$

where *T*^∗^ is SL interface temperature, the subscript *i* = *α*, *β*, *T_E_* is the eutectic temperature, *m* is the liquidus slope, *C_E_* is the eutectic concentration, Γ is the Gibbs–Thomson coefficient, *κ* is the SL interface curvature, *f* (*φ*, *θ*_0_) is representing the SL interface energy anisotropy, *φ* is the angle between the SL interface normal and the *x* axis, and *θ*_0_ is the angle of crystal orientation to the *x* axis.

In Equation (2), the coefficients of *T_E_*, *m*, *C_E_*, and Γ are physical parameters, shown in Table 1. The *κ* and *f* (*φ*, *θ*_0_) are calculated from solid fractions. For isothermal solidification, the SL interface equilibrium composition $C_{l}^{*}$ is the only unknown variable in Equation (2), which should be solved during each time step. The local actual liquid composition of each SL interface cell *C_l_* is updated to the local equilibrium composition $C_{l}^{*}$. In order to achieve mass conservation, a quantity of mass is provided, resulting in the increment of solid fraction ∆*f_s_*, which is governed by:

(3) $$\Delta f_{s,i}=(C_{l,i}^{*}-C_{l})/(C_{l,i}^{*}(1-k_{i}))$$

It can be seen that $C_{l}^{*}$ is the key parameter in SL interface kinetics; $C_{l}^{*}$ is a function of the SL interface curvature *κ* and the SL interface normal *φ*, as seen in Equation (2). So the numerical simulation of the SL interface curvature and SL interface normal determines CA model’s quantitative ability. In the present work, we used the height function method [24] to improve the accuracy of SL interface curvature calculation, which was implemented for the first time in the CA model.

The computational domain is divided into uniform rectangular cells. Each cell should be one of the six states: *α* SL interface, *β* SL interface, three phases SL interface, *α* solid, *β* solid, and liquid. The *α* solid state means a cell is full of *α* phase in solid state, and the *α* SL interface state is a mixture of *α* solid and liquid. Consequently, the three phases SL interface state is a mixture of *α* solid, *β* solid and liquid. Thus, the eutectic growth is the combination growth of all of the SL interface cells. The growth of *α* or *β* SL interface cells follow the same growth kinetic in Equation (3). The three phase SL interface cell allows the *α* and *β* phases to grow independently into the remaining liquid phase until it is full of solid *α* and *β* phases. Thus, the three phases SL interface cell state will change into the solid state. If the *α* solid fraction is greater than 50%, it is marked as *α* solid state; otherwise it will be changed into *β* solid cell.

**Figure 1:**
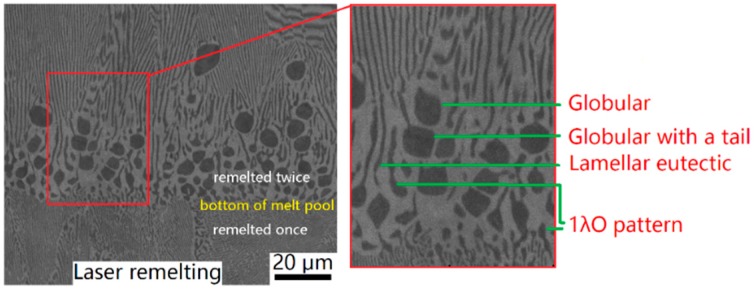
Backscattered electron images of anomalous eutectic morphologies at the bottom of melt pool after laser remelting Ni-30wt.%Sn powders twice, and its enlarged view of typical morphologies of anomalous *α*-Ni phase.

The experiments were designed by laser remelting twice on the Ni-30wt.%Sn alloy powders under the same process parameters, as seen in Figure 1. Laser power is 100 W, and two laser scanning velocities *L* used, 1.0 mm/s. The Ni-30wt.%Sn alloy powders were produced by plasma rotating electrode process (PREP) technology. Due to the powders having higher laser absorptivity and lower thermal conductivity, the melt pool that formed in the first remelting was larger than that in the second laser remelting process. The remelted specimens were cut along the transverse direction, which was vertical to the scanning direction of the laser beam. The benefit of laser remelting twice is that the fine lamellar eutectic from the first laser remelting would be the initial microstructure for the second laser remelting. A scanning electron microscope (SEM) of TESCAN, VEGAII-LMH, Brno, Czech Republic, was used to observe the microstructure.

## 3. Results

### 3.1. Anomalous Eutectic Morphologies

Figure 1 shows the anomalous eutectic morphologies observed in the laser remelting process. The dark phase is *α*-Ni, and the light phase is *β*-Ni_3_Sn. The *α*-Ni morphologies can be summarized into four types: globular, globular with a tail, lamellar eutectic, and 1*λ*O pattern, as shown in Figure 1. Compared to eutectic colony solidified from undercooled melt, the anomalous eutectic morphologies during laser remelting process were sandwiched between lamellar eutectic at the bottom of the melt pool after laser remelting Ni-30wt.%Sn powders twice, as seen in Figure 1. The microstructure evolution at the bottom of melt pool can be divided into two processes: one is the lamellar to anomalous transition (LAT), which indicates the anomalous growth from the lamellar eutectic substrate; the other is the anomalous to lamellar transition (ALT), after which, the final microstructure becomes coarser lamellar eutectic. The LAT process is beyond the expected epitaxial growth from the lamellar eutectic substrate. This is contrary to epitaxial dendrite growth from the dendritic substrate. The ALT process is also remarkable in that, as the solidification process continues, lamellar eutectic growth is more competitive than anomalous eutectic.

### 3.2. Temperature Filed at the Bottom of Melt Pool

Understanding of the temperature field evolution at the bottom of melt pool is the foundation to understanding the LAT and ALT processes during anomalous growth. Previous researches used a fixed cooling rate *R* = *GV* to investigate dendrite growth in the molten pool [25]. A constant cooling rate is appropriate for the thermal field at the top of the melt pool, where the growth velocity of the SL interface equals the laser scan speed. However, at the bottom of melt pool, the substrate is heated (melting) and cooled (solidifying), sequentially. The cooling rate *R* at the bottom of melt pool is obviously not constant.

**Figure 2:**
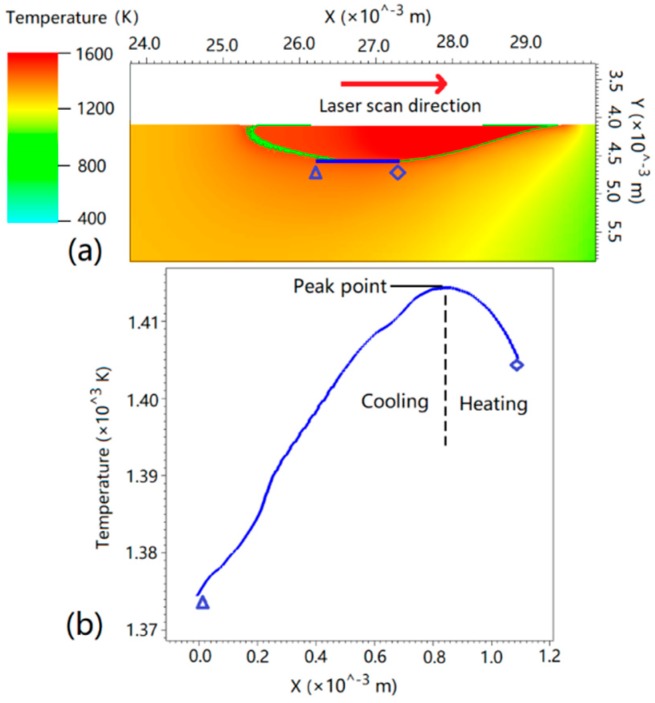
Numerical simulation of thermal field and lineout at the bottom of melt pool. (**a**) temperature distribution of melt pool; (**b**) the temperature lineout at the bottom of melt pool.

We simulated the steady state temperature field during laser remelting process by finite difference method. The longitude cross-section of the temperature field distribution is shown in Figure 2a. The parameters used in the simulation were *P* = 100 W, laser scan speed *V* = 1.0 mm/s, and laser radius *r_L_* = 2 mm. We selected a lineout along the *x* axis at the bottom of melt pool, the temperature distribution of which is shown in Figure 2b. It can be seen that the peak point represents the deepest position of the melt pool, on the right of which is heating and remelting, and on the left of which is cooling and solidifying.

According to the steady state temperature field in Figure 2b, the temperature field along the *x* coordinate at the bottom of melt pool can be approximated to a moving parabola, as seen in Equation (4).

(4) $$T(x,t)=a{\left(x-Vt\right)}^{2}+b,(a<0,b>0)$$

where *Vt* means the *x* position of peak temperature point at the center of laser beam, as seen in Figure 2b.

Based on Equation (4), the cooling rate *R* at the bottom of melt pool can be derived as:

(5) $$R=-\frac{\partial T}{\partial t}=-\frac{\partial [a{\left(x-Vt\right)}^{2}+b]}{\partial t}=2aV(x-Vt)$$

The cooling rate *R* at the bottom of melt pool is a linear function of time *t*. For a fixed position *x_m_*, when *Vt* < *x_m_*, the cooling rate is *R* < 0 (heating); when *Vt* = *x_m_*, *R* = 0; and when *Vt* > *x_m_*, *R* > 0 (cooling). So steady state directional solidification cannot describe the thermal condition here.

### 3.3. CA Simulation of Anomalous Eutectic Growth

The physical parameters were as previously described [26,27], as seen in Table 1.

They were modified for rapid solidification according to the Aziz model [28]. The details of the anomalous eutectic growth CA model: directional solidification of the regular eutectic was set as CA model’s initial condition. Due to the high energy input and absorption, which resulted in a high temperature gradient and cooling rate, the latent heat of solidification and the recalescence phenomenon were neglected in the present simulations. In the CA model, nucleations of *α*-Ni were simplified to an exponential function of undercooling. Due to the rules that govern *α*-Ni particulate’s orientation are unknown, each particulate was set as random for simplification. Actually, the simulation results were not influenced much by the nucleation orientations. We tried to priori set some unmelted *α*-Ni particulates with fixed orientations in the melt, and similar results were obtained, except that the priori set particulates grew larger than the nucleations. The temperature gradient, G, was fixed to 1.5 × 10^6^ K/m. The pulling velocity, *V*, was linearly increased from 0 µm/s to 2000 µm/s in 0.015 s representing a linearly increased cooling rate. The lamellar spacing in experiment was quite small, approximately 0.5 µm, as shown in Figure 1. And the curvature calculation in the present CA model needs at least several grids. So we used small mesh size of 0.01 µm to give quantitative results. Thanks to massively parallel GPU, the simulation finished within 10 h for 4,000,000 steps (≈0.015 s). The eutectic Ni-Sn alloy was used in the present simulations for a more general understanding of anomalous eutectic growth.

**Figure 3:**
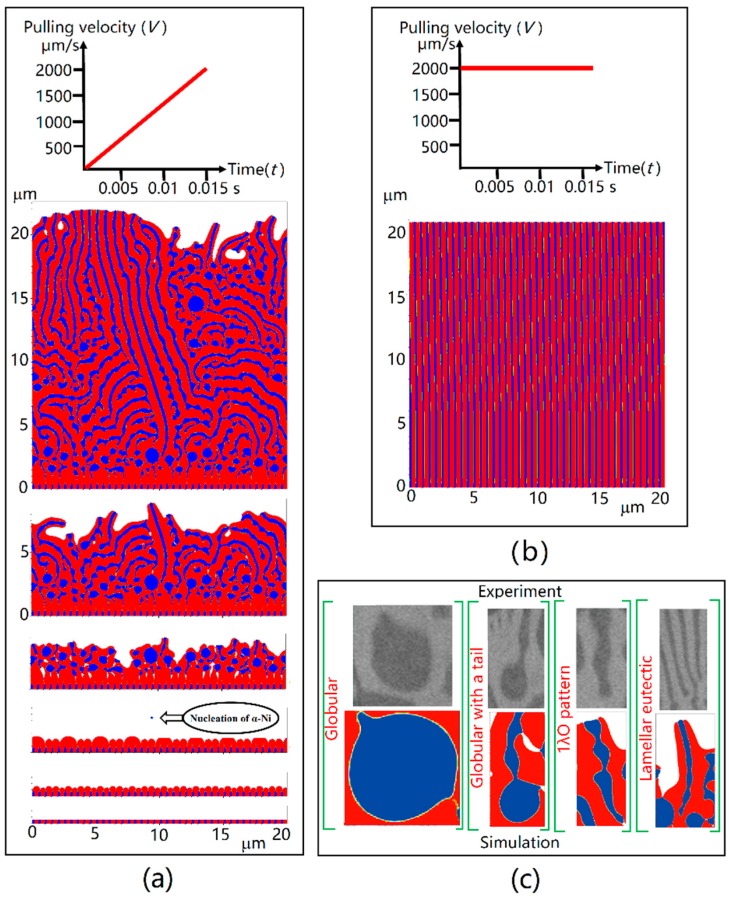
CA simulations of Ni-Sn anomalous eutectic growth under various cooling rates, and the comparison to experiment results: (**a**) temperature gradient *G* = 1.5 × 10^6^ K/m, pulling velocity *V* linearly increased from 0 µm/s to 2000 µm/s in 0.015 s; (**b**) temperature gradient *G* = 1.5 × 10^6^ K/m, pulling velocity *V* = 2000 µm/s; (**c**) typical anomalous eutectic morphologies of *α*-Ni.

The results of anomalous eutectic growth by CA simulations are shown in Figure 3a. Lamellar eutectic seeds were initialized to represent the substrate at the bottom of melt pool. Blue phase represents *α*-Ni; and red phase is *β*-Ni_3_Sn. It can be seen that *α*-Ni underwent overgrowth of *β*-Ni_3_Sn. After that, the growth of cellular *β*-Ni_3_Sn resulted in the enriched Ni solute in front of the SL interface. Nucleations of *α*-Ni occurred, as seen in Figure 3a. Therefore, the *α*-Ni phase was discontinuous at the bottom of melt pool, however the *β*-Ni_3_Sn phase was continuously grown upward, which agreed with the electron back-scatter diffraction pattern (EBSD) analysis [21].

We simulated steady state directional solidification with constant *V* = 2000 µm/s, which was at the scale of the laser scan speed, the result of which was epitaxial growth from the lamellar eutectic substrate, as shown in Figure 3b. That is, when the very fine regular eutectic grew at high cooling rate since the beginning, it displayed epitaxial growth from the lamellar eutectic substrate.

All of the four typical morphologies observed in experiments: globular, globular with a tail, lamellar eutectic, and 1*λ*O patterns have be seen in CA simulation results, as seen in Figure 3c. It is worthy to note that globular morphology with a tail is an intermediate state between globular and lamellar eutectic. The details of globular, globular with a tail, lamellar eutectic, and 1*λ*O pattern are shown in the experimental results Figure 1.

## 4. Discussion

The mechanism of the LAT process: *β*-Ni_3_Sn overgrows *α*-Ni and subsequently *α*-Ni particulates nucleate. Why is it not epitaxial growth from the lamellar eutectic substrate? The reason should be that the *α*-Ni phase has higher SL interface curvature undercooling than the *β*-Ni_3_Sn phase, because the volume faction of *α*-Ni in the eutectic is *f*_α_ = 0.318 [26]. The smaller the volume faction turns the higher the SL interface curvature undercooling. As we mentioned before, the lamellar spacing of the substrate was quite small, approximately 0.5 µm, which was solidified under rapid cooling rate. So when the very fine lamellar eutectic grows at a much smaller cooling rate, it is reasonable to observe the *α*-Ni overgrowth by *β*-Ni_3_Sn, as shown in Figure 3a. It is indicated that the rapidly changing cooling rate *R* has great influence on the growth of anomalous eutectic.

The mechanism of ALT process is the competitive between anomalous and lamellar eutectic. Some globular *α*-Ni nucleations were wrapped by *β*-Ni_3_Sn rapidly. Some α-Ni nucleations grew into lamellar eutectic coupled with *β*-Ni_3_Sn. The larger the *α*-Ni nucleation size, the more they tended to be were wrapped. The smaller *α*-Ni nucleations became the origin of lamellar eutectic. The growing size of *α*-Ni nucleations is proportional to the distance from the nucleation position to *β*-Ni_3_Sn SL interface. For instance, the first nucleation in Figure 3a was almost the largest *α*-Ni particulate, which was nucleated far from the *β*-Ni_3_Sn SL interface.

From the comparison in Figure 3c, the morphological agreement between experiments and CA simulations indicates that the anomalous eutectic evolution process generated by the present CA model is convincing. The changing of cooling rate is the key control parameter to the anomalous eutectic growth. The solidification in undercooled melt also had a rapidly changing cooling rate process due to the recalescence phenomenon. So we believe that the anomalous eutectic growth in undercooled eutectic melt follows similar mechanism as described in this article.

Zhao et al. [29] discovered that the microstructure during solidification of deep undercooled Ag-Cu melts has three regions: the anomalous eutectic close to the nucleation position, the coexisting region of the anomalous and lamellar eutectic, and the lamellar eutectic region. It is considered that the formation of anomalous eutectic is due to the partial remelting of the primary solidified lamellar eutectic. However, Mullis’s quantitative experimental analysis [17] shows that remelting-based anomalous growth models are infeasible, because the volume faction of the anomalous eutectic is much larger than the recalescence solid fraction. The solidification mechanism of the LAT and ALT in anomalous eutectic growth presented in this article explains the formation of large volume fractions of anomalous eutectic. It is also an evidence to Hogan’s proposal [30] that the anomalous eutectic was formed by repeated nucleation after overgrowth by the faster-growing phase.

## 5. Conclusions

During laser remelting of the Ni-Sn alloy, the thermal distribution at the bottom of melt pool is simulated by a finite difference method. A cellular automaton (CA) model is developed to simulated the anomalous growth. It is found that the cooling rate at the bottom of melt pool is a linear function of time. The present work shows that anomalous eutectic is an extreme situation caused by the instability of the lamellar eutectic under rapidly changing cooling rate conditions. The linearly increased cooling rate started from 0 at the bottom of melt pool had great significant on the formation of anomalous eutectic microstructure. The anomalous eutectic growth mechanism was divided into two processes: one is the lamellar to anomalous transition (LAT); the other is the anomalous to lamellar transition (ALT). Simulation results show that the mechanism of the LAT is that one phase overgrowing the other and subsequently nucleating of the other phase. The mechanism of the ALT is the competitive growth between anomalous and lamellar eutectic. The anomalous eutectic mechanism obtained in the present investigations well explains the anomalous eutectic growth at the bottom of melt pool during laser remelting process.

## Figures and Tables

**Table 1:** Thermal physical parameters of Ni-Ni_3_Sn eutectic alloys.

Constant	Value
Eutectic temperature (*T_E_*)	1403 K
Eutectic concentration (*C_E_*)	18.7 at.%
*α* liquidus slope at *T_E_* (*m_α_*)	−21 K/at.%
*β* liquidus slope at *T_E_* (*m_β_*)	37 K/at.%
*α* partition coefficient (*k_α_*)	0.57
*β* partition coefficient (*k_β_*)	1.21
Diffusion coefficient of solute (*D_l_*)	5.0 × 10^−9^ m^2^/s
*α* Gibbs-Thomson coefficient (*Г_α_*)	2.98 × 10^−7^ mK
*β* Gibbs-Thomson coefficient (*Г_β_*)	2.1 × 10^−7^ mK
